# New Appliances and Things Medical

**Published:** 1902-05-03

**Authors:** 


### NEW APPLIANCES AND THINGS MEDICAL.

[We shall be glad to receive at our Office, 28 & 29 Southampton Street, Strand, London, W.C., from the manufacturers, specimens of all new preparations and appliances which may be brought out from time to time.]

### THE "JAPA" SANITARY PAPER BLIND.

(Agents: REYNOLDS AND BRANSON, LIMITED, 13 BRIGGATE, LEEDS. MAKERS: HOWARTH AND CO., CHURCH STREET, HUNSLET, LEEDS.)

THESE sanitary blinds, which are intended to be used in place of the ordinary linen variety, are an invention which should appeal to the authorities of hospitals and other public institutions. They are made of stiff sanitary paper in three colours—red, blue, and cream. They are hemmed with strong thread top and bottom, so that they can be securely fixed to the roller and to the lath respectively. Further developments in these blinds are—1. Side hemming, so that they are practically untearable. 2. The attachment of handsome fringes to the bottom lath. In appearance these blinds are practically indistinguishable from linen blinds. They are more homogeneous and less pervious to light. They roll and unroll with extreme ease without creasing, and finally a new blind costs less than does a single washing of those of the ordinary linen. From a sanitary and hygienic point of view, we highly approve of the "Japa" blind, and believe that it has a great future, not only for use in public institutions, but in the windows of shops and dwelling houses.

### ALLENBURY'S LIQUID BEEF.

(ALLEN AND HANBURYS, LIMITED, BETHNAL GREEN, LONDON, E.)

ALLENBURY'S Liquid Beef is a new raw meat juice preparation which possesses dietetic properties little inferior to the fresh juice of meat prepared according to the usual domestic method. Tradition dies hard, and the almost universal belief that beef tea and meat extracts possess nutritive properties of value to the invalid is one of the most dangerous of the many fallacies which beset sick-room dietetics. The nutritive principles of meat are insoluble in boiling water. This is a fundamental fact which is known to every chemist and to every physiologist—any process therefore of meat extraction, or decoction which entails the application of heat, deprives the resultant liquid of all nutritive value. The extraordinary popularity of meat extracts is a striking testimony of the difficulty which the human mind experiences in making accurate deductions from observed facts. Because ninety-nine people out of a hundred feel invigorated and refreshed after a cup of beef tea, the incorrect deduction has been made that beef tea has supplied them with a source of energy or, in other words, that it is a food: so far from this being the case, beef tea is nothing more than the lash which flogs the weary horse, it is an instrument or stimulus for accelerating the expenditure of energy, and hence in the long run it has practically the same effect as alcohol, strychnine or ammonia, which can only tide us over a present difficulty, make us live, so to speak, on our capital, and anticipate our resources and physical strength. The liquid beef to which we have referred belongs to another category, the nutritive properties of the meat from which it is prepared have not been destroyed by heat, they are absolutely soluble, and in an essentially assimilable condition. The juice is concentrated and preserved without the addition of any objectionable antiseptic, it is pleasant to the taste, and is a true nitrogenous food, and absolutely adapted for the repair of tissues which are wasted by disease processes.

### ALGINOID OF IRON, THYROGLANDIN, SAVARESSE'S MEMBRANEOUS CAPSULES, CASCARA-HAWLEY.

(EVANS, LESCHER AND WEBB, 60 BARTHOLOMEW CLOSE, LONDON, E.C.)

MESSRS. EVANS, LESCHER AND WEBB have recently forwarded for our examination certain pharmaceutical preparations of great artistic merit. Alginoid of iron is a combination of alginic acid and iron, originally, we believe, suggested by the late E. C. C. Stanford as a substitute for the more usual preparations of iron. Its value as a therapeutic agent lies in the fact that ferric alginate is insoluble in the acid secretions of the stomach, and hence it cannot disturb the functions of this delicate organ; moreover, it is quite tasteless, slightly laxative, and very valuable in the treatment of anæmia. It is supplied by Messrs. Evans, Lescher and Webb in powder, or in 3-grain coated pills, two of which constitute an effective dose. Thyroglandin (Stanford) is a reliable preparation containing the active principles of the sheep's thyroid prepared according to process devised by the late Mr. E. C. C. Stanford. It is four times the strength of the fresh gland, and is supplied in powder or soluble coated pills containing one grain of the powder in each. Savaresse's membraneous capsules contain sandal wood or copaiva. They differ from the ordinary variety of capsules in that the casing or envelope is not composed by gelatine but of an animal membrane, which does not readily rupture in the stomach. Hence by taking these disagreeable drugs in the form of Savaresse's capsules the aromatic eructations which usually follow their ingestion are largely avoided. Cascara-Hawley, a speciality of Messrs. Evans, Lescher and Webb, is an admirable preparation of cascara sagrada in gelatine capsules by a special process of concentration. The ordinary B.P. dose is reduced in bulk and inserted in a capsule which can be readily swallowed by a child. Cascara-Hawley is a peculiarly active preparation of this well-known vegetable aperient, and is free from the griping effect of many of the crude preparations used by the public.

